# Profile of cannabis users among a population of Tunisian young adults residing abroad

**DOI:** 10.1192/j.eurpsy.2024.835

**Published:** 2024-08-27

**Authors:** M. Moalla, N. Charfi, S. Sellami, I. Gassara, R. Feki, N. Smaoui, S. Omri, L. Zouari, J. Ben Thabet, M. Maalej Bouali, M. Maalej

**Affiliations:** ^1^Psychiatry C, Hedi Chaker Hospital; ^2^Psychiatry C, Hedi Chaker Hospital of Sfax, Sfax, Tunisia

## Abstract

**Introduction:**

Tunisian emigrants may consume psychoactive substances in other countries. This community is exposed to sociocultural and legal contexts different from those in Tunisia and the degree of acculturation would tend to increase over time. However, data on the use of psychoactive substances, particularly cannabis, in this particular population are scarce.

**Objectives:**

This study aims to determine the profile of Tunisian young adults residing abroad who use cannabis.

**Methods:**

We conducted a cross-sectional, descriptive and analytic study. It was carried out in the form of an online survey. We focused on young Tunisians people who have completed their secondary studies at the pilot high school of Sfax and currently residing abroad. Data collection was through Google Forms administred questionnaire.

**Results:**

Thirty-five participants were included in our study. Cannabis use behavior affected 48.6% of them (N=17)) and it was done with friends in a festive setting in 88% of cases (N=15). Cannabis use was more common among people who were single (p=0.001), living alone (p=0.047), had a psychiatric history (p=0.032) and hanging out with friends who also smoked cannabis (p=0.032).Cannabis use was also more common among cigarette smokers (p=0.000) and alcohol consumers (p=0.000). It was significantly more common among people who shared erroneous beliefs about cannabis, that it is a mild drug (p= 0.024) and that it does not cause dependence (p= p=0.042).

**Conclusions:**

Cannabis use among Tunisian young adults residing abroad seems to be a form of poly-consumption, more common among singles, living alone and having a psychiatric vulnerability. These findings underscore the need for targeted interventions and educational initiatives to address cannabis use within this specific population.

**Disclosure of Interest:**

None Declared

